# Ten Years of the International Cancer Control Partnership: Promoting National Cancer Control Plans to Shape the Health System Response for Cancer Control

**DOI:** 10.1200/GO.22.00232

**Published:** 2023-01-11

**Authors:** Julie S. Torode, Zuzanna Tittenbrun, Yannick Romero, Sonali E. Johnson, Jean-Marc Bourque, Leslie S. Given, Karin E. Hohman, Ernest Hawk, Lisa M. Stevens

**Affiliations:** ^1^ Global Oncology Group, Institute of Cancer Policy, Kings College, London, United Kingdom; ^2^ Knowledge, Advocacy and Policy Team, Union for International Cancer Control, Geneva, Switzerland; ^3^ Radiation Oncology, Center Hospitalier de l'Universite de Montreal (CHUM), Montreal, Quebec, Canada; ^4^ Strategic Health Concepts, Arvada, CO; ^5^ Division of Cancer Prevention & Population Sciences, University of Texas MD Anderson Cancer Center, Houston, TX; ^6^ Programme of Action for Cancer Therapy, International Atomic Energy Agency, Vienna, Austria

## Abstract

Growing premature mortality because of cancer is an increasing public health concern in all countries. This article reviews 10 years of the International Cancer Control Partnership (ICCP) considering the themes of National Cancer Control Plan (NCCP) support, technical assistance, governance, and the renewed momentum of global calls to action. ICCP has provided key resources for the cancer community by hosting a portal with national cancer control and noncommunicable disease (NCD) plans, strategies, guidelines, and key implementation guides for a growing community of best practices. ICCP partners have responded to the changing needs of country planners, adjusting technical guidance as needs evolve from planning to implementation at the national level with an associated shift to peer-to-peer learning and knowledge exchange. The ICCP offer to assist countries in cancer planning continues to be relevant as countries focus on implementation of global initiatives for breast, cervical, and childhood cancers. These initiatives are important to drive priority actions and a systems approach in the emerging road map on NCDs—a message that will be supported by a second global review of NCCPs in 2023. This is critical for driving national action in all countries on cancer and other NCDs in line with global health commitments made for 2030 and adopted by the United Nations General Assemblies. ICCP sees robust systems and financial planning for implementation, monitoring, and evaluation of NCCPs and protection from cancer-related catastrophic expenditure, as critical to longer-term sustainability and success. ICCP calls for national policymakers to prioritize integration of cancer prevention and control into emerging universal health care approaches, including pandemic preparedness/health system resilience and calls for an equity focus in new NCCPs.

## INTRODUCTION

**FIG 1:**
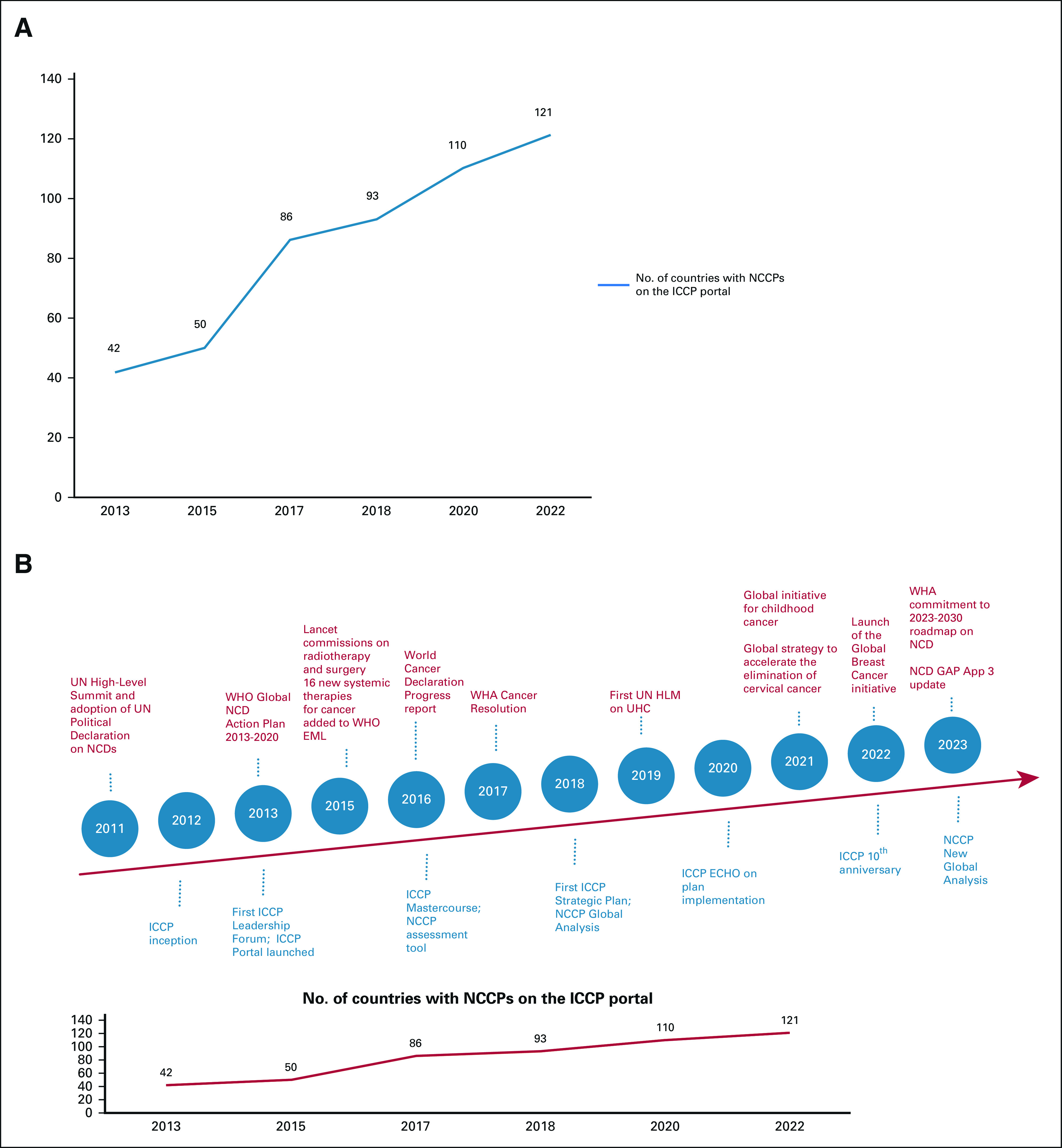
(A) No. of countries with NCCPs on the ICCP portal and (B) advocacy and policy timeline for cancer and other NCDs with key ICCP inputs and milestones. Global milestones from 2011 through 2023 (red) and ICCP milestones (blue). The increase in publicly available NCCPs on the ICCP portal over time. EML, essential medicine list; HLM, high-level meeting; ICCP, International Cancer Control Partnership; NCCP, National Cancer Control Plan; NCD, noncommunicable disease; UHC, Universal Health Coverage; UN, United Nations; WHA, World Health Assembly.

In May 2022, the World Health Assembly (WHA)—a convening of Ministers of Health of all WHO Member States—committed to developing a roadmap on noncommunicable diseases (NCDs) to drive individual, national momentum toward the 2030 global targets expressed in the NCD Global Action Plan^1^ and implementation of the full suite of best buys in an integrated approach to the Sustainable Development Goals (SDGs) and Universal Health Coverage (UHC; Fig 1). More than 10 years ago, the global cancer community was similarly preparing for a critical WHA, one that initiated the development of the framework of nine targets and 23 indicators that form the basis of global NCD commitments.^2^ Cancer advocates fought hard for the United Nations (UN) High Level Meeting in 2011 (Fig 1) and were pushing for engagement across the full continuum of care during dialogues. The concern was that the focus could remain on NCD prevention only and perhaps overlook critical actions in cancer prevention, early detection, diagnosis, treatment, and palliative care entirely, with the potential for leaving patients with cancer behind.^3,4^

**CONTEXT**

**Key Objective**
Does a decade of advocacy on National Cancer Control Plans (NCCPs) provide the necessary policy and technical support to drive national cancer prevention and control efforts?
**Knowledge Generated**
A decade of the international cancer control partnership highlights that a multiparter platform can host a one stop shop (the International Cancer Control Partnership [ICCP] portal) of NCCPs and associated resources with flexible technical assistance as needs evolve. ICCP is a learning collaborative that has led to more NCCPs in the public domain, more interaction between cancer experts and Member States, and a channel for coordinated engagement between national implementers and international partners.
**Relevance**
ICCP is a navigator to priority actions, resources, and technical assistance when refreshing NCCPs for the final 5-year push (2025-2030) toward targets expressed in key global action plans and initiatives. The clinical community in low- and middle-income countries should harness this opportunity to leverage NCCPs for accelerated uptake of national cancer guidelines, workforce reforms, financial sustainability, and accountability (N = 150).

The sense of urgency of advocates during the High-Level Meeting was driven by the projected increase in global cancer burden, with the most rapid rates of increase expected in low- and middle-income countries (LMICs). In 2022, those projections are proving to be real with the percentage of premature mortality globally because of NCDs now at 74%, versus 63% in 2008.^5-7^ In addition, cancer is now the leading cause of premature death in 48 countries and the second leading cause of premature death, after cardiovascular disease, in a further 43 countries.^8^ The critical role of advocacy in driving national policy and implementation of cancer plans therefore persists, especially as we look to strengthen recovery and health systems resilience after the COVID-19 pandemic.^9^

## BACKGROUND OF INTERNATIONAL CANCER CONTROL PARTNERSHIP

Despite advocating for National Cancer Control Plans (NCCPs) in 2012, funding for their development remained scarce. Engagement with countries was therefore sporadic and fragmented, without the continuity needed for such a complex activity. The International Cancer Control Partnership (ICCP) was born out of a desire to advance NCCP development and to coordinate efforts among global entities in collaboration with countries. Cofounded by the US National Cancer Institute and the Union for International Cancer Control, after a roundtable meeting at the World Cancer Congress in Montreal in 2012 (Fig 1), this network of now 21 organizations shares a common vision that all countries are implementing a quality cancer control plan. The NCCP, as recommended by the WHO, is linked to national NCD control efforts and integrated into UHC and the broader SDGs agenda.^10^ Since the founding of the ICCP, we have evolved into a learning collaborative that supports the agency of countries not only to develop but also to implement their own context-specific NCCPs. This article shares the story of the evolving partnership and its readiness to adapt to support country- and global-level efforts to advance health systems for cancer control. Key elements of its success include (1) facilitating cross-stakeholder engagement at the country level; (2) alignment with initiatives led by UN agencies, in particular WHO, International Atomic Energy Agency, and International Agency for Research on Cancer for consistency with UN messaging and leveraging of emerging cancer initiatives; (3) extension of ICCP to include partners across cancer disciplines with implementation experience in LMICs and connectivity with a large network of expert contributors, including regional and local perspectives; and (4) strong partnership through robust governance and pan-partner implementation. A persistent challenge throughout the past decade is that building the case for financing for implementation of NCCPs is difficult at the national level and political will for prioritization for cancer prevention and control still lags behind other areas of public health concern.

## SUPPORT TO DRIVE NATIONAL CANCER CONTROL PLAN DEVELOPMENT

At the time ICCP was established, one of the key resources for countries first setting out to develop cancer plans was the structure and content of other NCCPs.^10^ In 2012, there were few such documents in the public domain. ICCP therefore set out to establish and keep current a repository of publicly available, national and regional cancer control plans, NCD plans, and any supporting resources, including WHO cancer country profiles. Our first effort identified 42 countries with NCCPs (mainly high-income countries). The portal now has a library of NCCPs from 121 countries (Fig 1).

In parallel, ICCP looked to more targeted efforts to support LMICs in initiating or enhancing national cancer control planning and implementation efforts. First, ICCP worked collaboratively to develop and implement the International Cancer Control Leadership Forums (ICCLF). Over the course of 5 years, a series of nine forums^11^ took place, supporting 38 countries in total. Each forum was regional in focus, identifying common topics with participating countries to shape the agenda. Country teams (composed of Ministry of Health/government, academic, and nongovernmental organization participants) developed their individual action plans during a 3-day meeting, while also building connections and collaboration across regions. The ICCLF, and subsequent mentorship to implement the action plans over the following 12 months, led to stronger country ownership across national stakeholder groups and leadership in cancer control.^12^ For example, following the Central Asia ICCLF, Uzbekistan's government allocated funds to strengthen cancer care delivery at the National Cancer Research Center, a key ICCLF implementing partner. The work also enabled ICCP to identify priority areas for future technical assistance through the ongoing relationships with national implementers.

## ADAPTING THE TECHNICAL ASSISTANCE RESPONSE AS NATIONAL NEEDS EVOLVE

The ICCLF focused on establishing NCCPs and the accompanying governance mechanisms, as well as collaboration steps aimed at implementation of priorities from the plans. By 2018, the partners realized that the content and quality of published NCCPs were highly varied, including with respect to their ability to shape and drive implementation. Therefore, in 2018, ICCP conducted a first global review of NCCPs and other cancer-related documents (Fig 1).^13^ ICCP coordinated review of more than 500 documents from 158 countries. Outreach directly with Member States encouraged sharing of draft NCCPs (almost ready for publication) to be included in the review.

The purpose of the review was to understand the extent to which current NCCPs aligned with a set of predefined, evidence-informed domains.^14^ As reported, plans were collectively strong on cancer prevention and early detection but limited on health systems response for treatment and palliative care, locally relevant research agenda setting, and workforce planning. In addition, costing and financial planning for their implementation were rarely addressed. The review has led to further research on specific areas of the NCCP, such as access to (1) essential medicines showing that NCCPs promote more effective access to medicines policies and identified policy deficits that together with patient financial protections could improve access^15^; (2) pathology that identified that only 14% of countries have included pathology and laboratory medicine in their national strategies^16^; and (3) radiotherapy revealing that countries have more machines per 100,000 population when this service is delineated in plans.^17^ Further manuscripts in preparation on palliative care and survivorship in Africa demonstrate a paucity of survivorship indicators in NCCPs.

The strengths of the ICCP were leveraged in this comprehensive review, working with a total of 68 cancer control experts across the globe, using a weighted questionnaire for a consistent approach across the core domains of an NCCP.^14^ The domains are as follows: introduction and overview; prevention; diagnosis, staging, and screening; treatment; palliative care and survivorship; service delivery; governance; health workforce; research; health information systems; finance; and overall summary. The same validated tool continues to be used by the ICCP Technical Assistance Group to respond to requests for NCCP review and provide country teams with individualized feedback in a standardized manner. After the first global review, technical assistance requests from countries increased, with asks ranging from ICCP partners serving on technical working groups in support of country efforts, review of new iterations of NCCPs, and provision of targeted experts to complement local NCCP teams and support implementation and evaluation efforts. A growing roster of expert reviewers benefit from mentoring by ICCP and vice versa, adding value to the technical assistance as representation and experience from LMICs increase. The technical assistance offer has, therefore, matured and diversified from simple review of a draft plan, to use a common assessment tool to both guide and provide consistency in reviews and to enhance the quality and implementation of the NCCP and drive priority actions in cancer control.^18^

## STRONG GOVERNANCE FOR STRONG PARTNERSHIP

As the number of partners grew, the need for targeted, collective, and measurable action was recognized. An ICCP strategic plan was developed, starting in 2014 (at the World Cancer Leaders' Summit, Mexico City [Fig 1]). The Strategic Plan included three main goals (1) to coordinate and leverage partner efforts to increase global support, political will, and country resources for development and implementation of NCCPs; (2) to provide guidance to countries as they strengthen their capacity to develop, implement, and evaluate NCCPs; and (3) to maintain and enhance the ICCP as a credible source of resources, guidance, and leadership in national cancer control planning.^19^ The operational structure includes a Steering Committee, three partner-led work groups (technical assistance, communications, and evaluation), and coordination of regular partner meetings. Partnership activities are built around three core principles: strong governance for harnessing the strengths of each ICCP partner; maintaining agility in our response to requests for technical assistance as national needs evolve; and ensuring alignment with global initiatives for shaping cancer-specific guidelines and national strategies. From an advocacy perspective, the last principle provides opportunities to harness political will and financing opportunities. For example, the tools and approaches from the ICCLF series were showcased at the World Cancer Leaders' Summit in Paris in 2016, linked with launch of the NCCP for France by French leaders and paired with a further opportunity for direct capacity building with a wider set of stakeholders through a Master Course during the World Cancer Congress in Paris that followed (Fig 1).

The strength of ICCP is illustrated by the growth in numbers of partners to 21 in 2022. These partners are governmental and intergovernmental, nongovernmental organizations, professional societies, and academic institutions (see Appendix 1 for the current list). The retention of partners is driven by the value of regular exchange with like-minded organizations and the opportunity for collaboration between partners in support of country-driven policy implementation to ensure that countries have an NCCP that can be and is being implemented. A recent survey of the members (internal report) revealed that up to four different ICCP members collaborated in support of any one country initiative.

## GLOBAL INITIATIVES FOR HARNESSING POLITICAL WILL AND FINANCING OPPORTUNITIES

Since its inception in 2012, ICCP has strived to link its work to key global health initiatives such as the SDGs,^20^ the WHA Global Cancer Resolution,^21^ and the Global Initiative for Cancer Registration.^22^

In the early years of the ICCP, partners noted that cancer was not often a primary focus when NCDs were discussed on the global stage. The strong NCCP focus of ICCP has stimulated not only national-level action but also internal and civil society advocacy to secure pan-Member State support to ensure that cancer control is included in national NCD strategies with planning and policy coherence across NCDs and other allied areas of health. This is illustrated in the most recent WHO report on cancer, which articulates where progress has been made and where weaknesses remain.^23^ As implementation readiness has improved and cancer programs have matured, the needs of cancer leaders and implementers internationally have also evolved. This has been the case as new global initiatives have emerged, such as the ambition to eliminate cervical cancer as a global health problem,^24^ the Global Childhood Cancer Initiative,^25^ the Global Breast Cancer Initiative,^26^ and Rays of Hope.^27^

**FIG 2:**
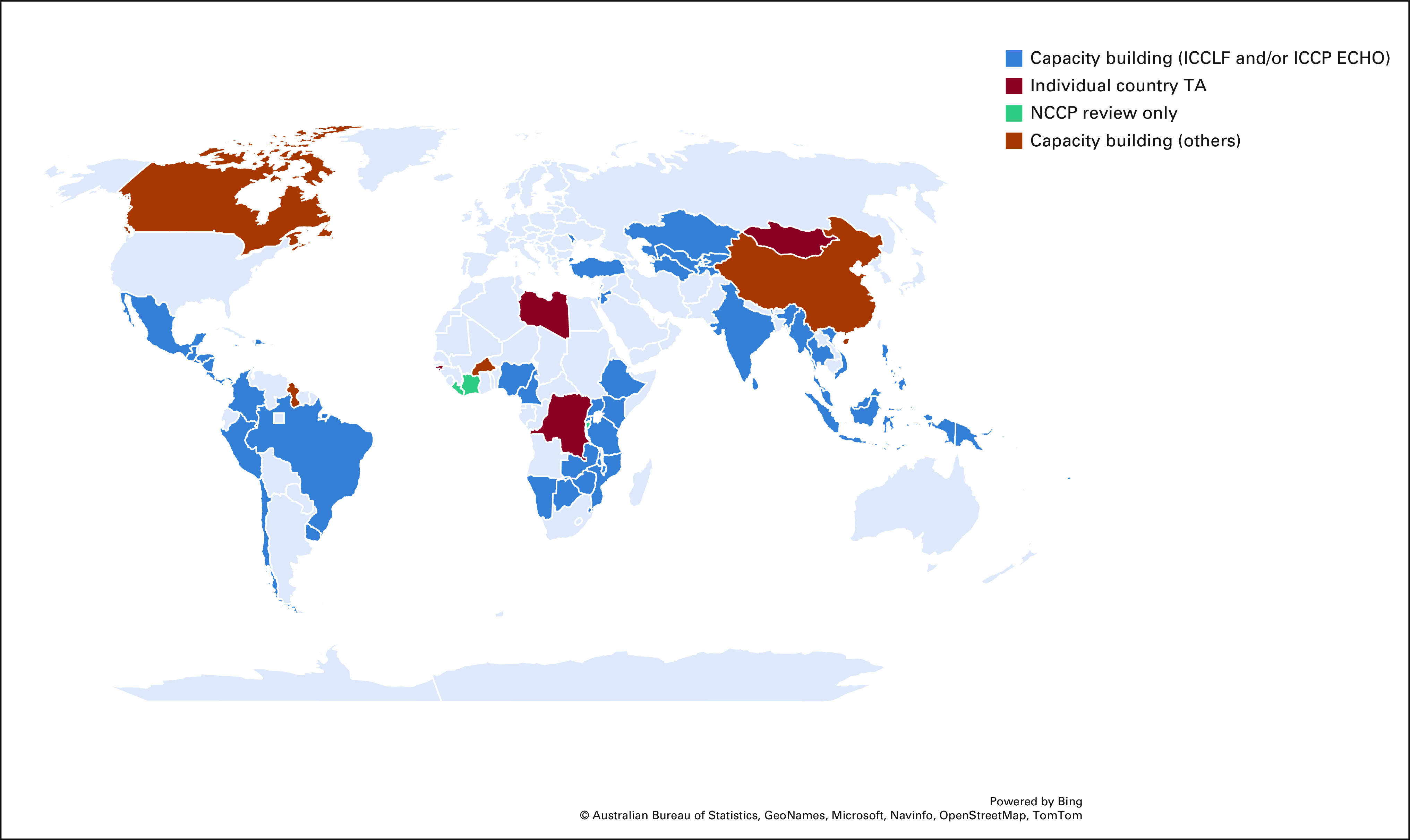
ICCP TA map. Types of TA are shown as capacity building (blue), individual country TA (red), review of NCCPs (teal), and other types of capacity building (orange). ICCLF, International Cancer Control Leadership Forums; ICCP, International Cancer Control Partnership; NCCP, National Cancer Control Plan; TA, technical assistance.

Leveraging the momentum created by these global priorities and recognizing the need for more focus on implementation to further progress cancer control, a complementary technical assistance offer, the ICCP ECHO, was established. The program consists of a peer-to-peer knowledge exchange for interested countries implementing their NCCPs.^10^ The virtual community of practice uses the Project ECHO^28^ technology–enabled collaborative learning model.^29^ In the first 2 years (2020-2022), 11 country teams from two world regions (Africa and Asia; Fig 2) have benefited from the bidirectional learning environment, which was able to continue throughout the COVID-19 pandemic and promote useful publications and resources that supported the pandemic response from a cancer perspective. Building on the approach from the ICCLF and the experience of convening regional ECHOs for cancer control, led by the National Cancer Institute,^30^ the curriculum focuses on evidence-based NCCP implementation strategies informed by topics identified by participants as key areas of need for implementation. Areas of interest include, but are not limited to, scaling up of cancer prevention and control services and costing of services for improved financial sustainability. In addition, program outcomes are measured, which assist in tracking knowledge acquisition and understanding contextual considerations that affect and inform NCCP implementation.^29^ Country progress in implementing the NCCPs is being monitored to be able to assess the long-term impact of this engagement.

Today, the ICCP portal includes NCCPs from 121 countries.^10^ ICCP has contributed to this public accountability through regular requests to Ministries of Health to post their plans and promotes these new additions to the portal. As new global initiatives are translated into national plans, these will also be captured. For example, of particular interest currently is the best practice exchange on breast and cervical cancer guidelines.

ICCP was established to inspire communication, coordination, and collaboration between organizations and countries working to improve cancer control planning. This is now a participatory activity, with engagement in countries across all WHO regions (Fig 2). Most countries do have NCCPs and will be refreshing them in 5-year cycles. Those being updated should reflect local contexts and needs and be the drivers for progress toward the countdown to 2030. ICCP partners are starting to see a shift, for example, toward more integrated approaches, particularly at the primary health care level and diagnostic and laboratory needs.

The ICCP partnership plays an important role in facilitating these broader dialogues at a global level, setting out the landscape of cancer control and its interconnectedness with other key agendas, including those related to NCDs, UHC, access to medicines, and maternal and child health. In this respect, the ICCP aims to grow in its role as a convenor of stakeholders and bring organizations and countries together for advocacy on evidence-based cancer control and in the sharing of planning tools and approaches. A priority is to broaden ICCP membership, involving partners from a wider range of countries and disciplines. In this respect, the ICCP is planning to expand peer learning opportunities through the ICCP ECHO program to encourage increased engagement and collaboration across regions.

In our ongoing efforts to promote the impact of NCCPs on health outcomes and health system strengthening, a second global NCCP review is planned in 2023. This includes the full set of NCCPs published to date and will assess current priorities around the world compared with 2018's baseline study, provide feedback to Member States to reflect how plans have evolved, and give a first indication to the extent to which areas of focus with commitments by the year 2030 (eg, UHC, equity, global initiatives, and emergency preparedness) are being adopted into national implementation plans.

In cancer, persisting inequities lead to unfair and avoidable differences in access to cancer services, quality of care, and outcomes. Millions of people around the world do not receive reliable information about behaviors that increase the chance of getting cancer. They are neither able to benefit from early detection of disease nor receive timely and adequate treatment and support if a cancer is diagnosed. These are the levers for change that NCCPs and policymakers need to address to improve cancer outcomes and reduce the social and economic impact of cancer on individuals, families, and communities. ICCP has refreshed its mission statement to include the cross-cutting issue of equity of availability and access to cancer information and services. We aim to increase understanding on how NCCPs can best reflect the equity imperative and work as a partnership to ensure that these learnings lead to addressing of cancer inequities at local and international levels.

In conclusion, as demonstrated in this article, ICCP has remained committed to supporting countries and global efforts to advance cancer control through a multilevel approach in its first decade. ICCP's collaborative approach with local-, national-, and global-level actors, including UN agencies, continues building on the central resource of NCCPs. ICCP-convened communities of practice foster bidirectional learning to advance the field by promoting adoption of evidence-based cancer control policies, adaptation of policies to respond to changing local needs, and innovations learned from country-level implementation. The partners have bilateral experience in supporting countries in the development, review, and implementation of NCCPs. In addition, several technical assistance offerings have included regional groups of countries and groups of countries from different world regions learning from each other and sharing challenges and best practices. A manuscript in preparation will provide detailed insights and case studies with specific examples of national impact of both the ICCLF and responsive technical assistance. These combined references will inform ICCP's next decade as we continue to adapt and grow alongside the global cancer community.

## APPENDIX 1

This article is written on behalf of the current partners:

1. African Organisation for Research and Training in Cancer (AORTIC)
2. American Cancer Society (ACS)
3. American Society of Clinical Oncology (ASCO)
4. American Society for Clinical Pathology (ASCP)
5. Canadian Partnership Against Cancer
6. Centers for Disease Control and Prevention USA (CDC)
7. ECHO Institute—University of New Mexico
8. Global Focus on Cancer
9. International Atomic Energy Agency (IAEA)
10. International Agency for Research on Cancer (IARC)
11. International Association for Hospice & Palliative Care (IAHPC)
12. International Society of Nurses in Cancer Care (ISNCC)
13. Jhpiego
14. Latin American and Caribbean Society of Medical Oncology (SLACOM)
15. North American Association of Central Cancer Registries (NAACCR)
16. National Comprehensive Cancer Network (NCCN)
17. The University of Texas MD Anderson Cancer Center
18. Two Worlds Cancer Collaboration
19. Union for International Cancer Control (UICC)
20. University of Hawaii. John A. Burns School of Medicine, Cancer Council of the Pacific Islands
21. US National Cancer Institute
